# Physicochemical Characterization and Finite Element Analysis-Assisted Mechanical Behavior of Polylactic Acid-Montmorillonite 3D Printed Nanocomposites

**DOI:** 10.3390/nano12152641

**Published:** 2022-07-31

**Authors:** Maria-Eirini Grigora, Zoe Terzopoulou, Konstantinos Tsongas, Dimitrios N. Bikiaris, Dimitrios Tzetzis

**Affiliations:** 1 Digital Manufacturing and Materials Characterization Laboratory, School of Science and Technology, International Hellenic University, 57001 Thessaloniki, Greece; megrigora@ihu.edu.gr (M.-E.G.); k.tsongas@ihu.edu.gr (K.T.); 2 Laboratory of Polymer Chemistry and Technology, Department of Chemistry, Aristotle University of Thessaloniki, 54124 Thessaloniki, Greece; terzoe@gmail.com (Z.T.); dbic@chem.auth.gr (D.N.B.)

**Keywords:** poly(lactic acid), montmorillonite, nanocomposites, Fused Filament Fabrication, nanoindentation, compression, FEA

## Abstract

This work aims to improve the properties of poly(lactic acid) (PLA) for future biomedical applications by investigating the effect of montmorillonite (MMT) nanoclay on physicochemical and mechanical behavior. PLA nanocomposite filaments were fabricated using different amounts of MMT (1.0, 2.0, and 4.0 wt.%) and 2 wt.% Joncryl chain extenders. The 3D-printed specimens were manufactured using Fused Filament Fabrication (FFF). The composites were characterized by Gel Permeation Chromatography (GPC), Melt Flow Index (MFI), X-ray Diffraction (XRD), and Fourier-transform infrared spectroscopy (FTIR). The thermal properties were studied by means of Differential Scanning Calorimetry (DSC) and Thermogravimetric Analysis (TGA). Moreover, the hydrophilicity of the PLA/MMT nanocomposites was investigated by measuring the water contact angle. The mechanical behavior of the PLA/MMT nanocomposites was examined with nanoindentation, compression tests, and Dynamic Mechanical Analysis (DMA). The presence of Joncryl, as well as the pretreatment of MMT before filament fabrication, improved the MMT distribution in the nanocomposites. Furthermore, MMT enhanced the printability of PLA and improved the hydrophilicity of its surface. In addition, the results of nanoindentation testing coupled with Finite Element Analysis showed that as the MMT weight fraction increased, as well as an increased Young’s modulus. According to the results of the mechanical analysis, the best mechanical behavior was achieved for PLA nanocomposite with 4 wt.% MMT.

## 1. Introduction

In recent years, nanoclays have attracted great interest as reinforcements for polymer matrices due to their ability to dramatically improve the physical, thermal, and mechanical behavior of polymers, even with small loadings [1]. Montmorillonite (MMT) is one of the most frequently used reinforcing inorganic nanofillers. It is a layered aluminosilicate clay, which is abundant in nature and has an inner octahedral layer of aluminum oxide/hydroxide, which is between two tetrahedral silicate layers. MMT exhibits many attractive properties that led to its widespread use as a filler, including its low cost, large surface area, electrical conductivity, cation exchange capacity, good gas barrier, biocompatibility, and flame retardancy [2], as well as its ability to improve the crystallization of polymers.

Nanocomposites based on polymer matrices with superior properties, such as thermal, electrical, and mechanical, have been studied extensively during the last decades [3]. In today’s green material research, nanocomposites based on nanoclays have a lot of potential as a small amount of nanoclay in the polymer matrix can enhance the mechanical and material behavior without losing processability [4]. Nanoclays can improve the mechanical properties, crystallization, and thermal stability of polyesters, including poly(lactic acid) (PLA) [5,6,7,8,9,10,11].

PLA is a thermoplastic polyester that is currently leading the market of biobased polymers. As a relatively inexpensive renewable material that can be 3D printed, it became popular fast, and its biocompatibility resulted in a wide range of biomedical applications such as tissue engineering [12,13]. However, PLA exhibits some limitations such as brittleness, poor thermal stability, very slow crystallization and biodegradation rates, low heat-distortion temperature, limited drawability, and low mechanical properties [6,14,15,16]. However, these characteristics can be overcome using nanofillers [3]. Nanofillers can restrict the chain movements of PLA, which improves PLA’s response to temperature change; thus, better shape recovery and higher elastic modulus values [4,17,18], and the dispersion of nanofillers is the most effective way to reinforce PLA [17]. The enhanced properties are imparted usually using only a small amount of nano-additives such as clays, which are typically ≤5 wt.% [7]. The printability of PLA has also been reported to improve [8]. Filled materials seem to be a better fit for 3D printing at moderate and high speeds than neat materials [9]. Nieddu et al. [19] noticed that PLA nanocomposites showed an improvement in the modulus as a function of the type and the content of clay compared with the pure polymer. The addition of organomodified montmorillonite (OMMT) with 5 wt.% enhanced the mechanical and thermal behavior of polymers as well as the processibility of PLA-based and TPU-based composites [20]. Moreover, PLA-based blends with a small loading of nanoclay (1 to 5 wt.%) and Joncryl (0.3 phr) are extremely interesting in the case of 3D printing technologies [21]. However, Othman et al. [22] noticed that the optimal mechanical properties for films were obtained in the case of PLA reinforced with 3 wt.% concentration of MMT. Bigger nanoclay loadings (≥5 wt.%) led to a reduction in the mechanical behavior of the films due to agglomeration. Makwakwa et al. [23] managed to improve the dispersion of hydrophilic Boehmite nanoparticles in a hydrophobic PLA matrix using chain extension and branching. As a result, the mechanical behavior of the developed nanocomposite material was improved. Furthermore, Coppola et al. [24] studied two different types of PLA reinforced with Cloisite 30B and using 3D printing, and they reported that PLA/clay filaments increased in the storage modulus at 35 °C, and according to TGA and DSC measurements, the nanoclays increased the thermal stability and acted as heterogeneous nucleating agents.

Fused filament fabrication (FFF) is a 3D printing method that involves the addition of polymeric material layer by layer to create a final 3D printed product [6]. The FFF process requires certain processability parameters that affect both filament fabrication and layer deposition during printing. Polymers are the most used feedstock in 3D printing out of a variety of possible materials. The use of 3D-printed polymers for final product production is rare nowadays due to their poor mechanical behavior, as well as due to poor electrical and thermal properties [10]. Therefore, reinforced polymers could be potentially used instead to overcome those disadvantages. Polymer nanocomposites have been widely studied in FFF [11,25,26], and it is expected that 3D printing to be used a lot in the biomedical applications sector [27]. PLA is 3D-printable; however, it might suffer from thermal degradation during processing. Significant efforts have been made to improve PLA performance by raising its molecular weight using a solid state or chain extension [12]. To overcome such an issue, chain extenders have been added during the preparation of the filament, with success leading to improvements in melt strength by promoting polymeric chain entanglement and limited degradation [28]. In a previous study, we developed PLA filaments using a multifunctional chain extender (Joncryl ADR^®^ 4400), and the addition of 2 wt.% Joncryl to PLA successfully enhanced the molecular weight, melt flow index, complex viscosity, and improved the printability and the mechanical behavior of PLA [29]. The presence of 2 wt.% Joncryl was sufficient to minimize the thermal degradation of PLA during processing and increased the average molecular weight from 81,700 g/mol to 124,300 g/mol.

This work aims to investigate the effect of MMT on the extrusion of PLA filaments and their subsequent 3D printing. Prior to their mixing and extrusion, an amount of MMT was prepared using the freezing/thawing-ultrasonic exfoliation method to improve the dispersion of MMT in the nanocomposites. The chain extender Joncryl was used to enhance the processibility of PLA/MMT filament fabrication by enhancing clay dispersion [30]. The use of MMT as a nanofiller is expected to improve the mechanical behavior and the printability of PLA and enhance the hydrophilic nature of the 3D-printed specimens’ surface for better performance in biomedical applications. Various physicochemical tests such as GPC, MFI, XRD, FTIR, contact angle, and thermal analysis were implemented to characterize the nanocomposite materials. Compression tests were also performed to evaluate the mechanical performance of 3D-printed structures. Moreover, nanoindentation tests were conducted to investigate the nanomechanical behavior of PLA nanocomposites. These tests were assisted by a finite element analysis (FEA) process to fit the nanoindentation load–depth curves and extract the nanocomposite materials’ stress–strain behavior. Such data are very scarce in the literature for the materials under study. The novelty of this study is the direct extrusion of materials without any prior pellet fabrication and the use of different methods to investigate and analyze the mechanical behavior of PLA/MMT nanocomposite filaments, using Joncryl and MMT as fillers in injection molding-grade PLA. The result is the fabrication of nanocomposite filaments with enhanced printability and mechanical properties suitable for 3D printing.

## 2. Materials and Methods

### 2.1. Materials

**Figure 1:**
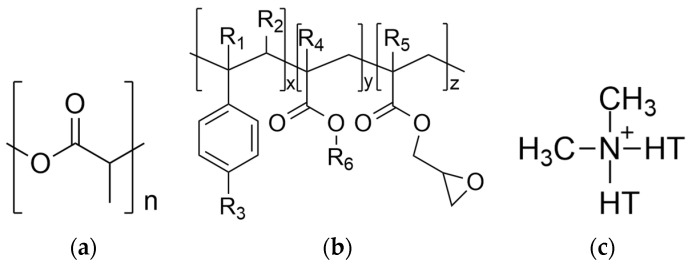
Chemical structures of (**a**) PLA, (**b**) Joncryl and (**c**) modified of Cloisite^®^ 20A.

In this study, poly (lactic acid) (PLA-Ingeo^TM^ Biopolymer 3052D, NatureWorks, Plymouth, Minnesota) was used as the polymer matrix. PLA 3052D was kindly gifted by Plastika Kritis S.A. (Iraklion, Greece) in the form of solid flakes with M_n_ = 81,700 g/mol (GPC) and is designed for injection-molding applications. Table 1 presents some of the properties provided by the supplier. The reactive agent Joncryl ADR^®^ 4400 was purchased by BASF (Ludwigshafen, Germany). It has an epoxy equivalent weight of 485 g/mol and a weight-average molecular weight of 7100 g/mol. In this work, the term “Joncryl” is used to represent Joncryl ADR^®^ 4400. Cloisite^®^ 20A (MMT) was supplied by Southern Clay Products (Gonzales, TX, USA). Cloisite^®^ 20A is modified with dimethyl, dehydrogenated tallow quaternary ammonium. In this paper, the term “MMT” is used to represent the Cloisite^®^ 20A, and the term “PLA” will be used to simplify and represent the term “PLA/Joncryl”. The chemical structures of PLA, Joncryl, and MMT are shown in Figure 1a–c [31,32].

### 2.2. Preparation of PLA/MMT Nanocomposite Filaments

Silica hydrophilic/ionic inorganic-based nanoparticles are particularly difficult to disperse uniformly in PLA [33]. For this reason, an appropriate amount of MMT was exfoliated using the freezing/thawing-ultrasonic exfoliation method prior to its mixing and extrusion using a modified approach based on the work of Chen et al. [34]. This technique not only will prevent the agglomeration of MMT nanoparticles but will also improve the dispersion in the PLA matrix. A small amount of MMT was immersed in ultrapure water and was magnetically stirred at 650 rpm for 24 h at room temperature. Afterward, it was mechanically mixed for 30 min using a T10 basic ULTRA-TURRAX^®^ Homogenizer from IKA (Staufen, Germany). Finally, it was ultrasonicated for 20 min until no aggregates were visible in the dispersion, using a UP100H Ultrasonic Processor (0.8 cycles, 80% amplitude) from Hielscher (Teltow, Germany) [34]. Then, the MMT suspensions were frozen and dried using a freeze drier (Scanvac, Coolsafe 110-4 Pro, Labogen, Scandinavia) for 10 days to obtain dried nanoparticles of MMT. Furthermore, prior to filament extrusion, Joncryl was milled into powder using a Thomas milling machine (Thomas Scientific, Swedesboro, NJ, USA) for 5 min, and all of the materials, including the PLA flakes, were dried under vacuum at 40 °C overnight.

Three different types of nanocomposite filaments were fabricated using PLA as the polymer matrix, Joncryl 2 wt.% as a chain extender, and MMT nanoclay as a reinforcement. Based on the results of our previous work [29], the addition of 2 wt.% Joncryl increases the molecular weight and improves the printability of PLA filaments. Three different contents of MMT (1.0, 2.0, and 4.0 wt.%) were added to the PLA filaments. The dried PLA, 2 wt.% Joncryl powder, and the predetermined amount of MMT were manually mixed by stirring and then placed into the hopper of a desktop filament extruder (Filament Maker-Composer 350, 3devo, Utrecht, The Netherlands), and each nanocomposite PLA/MMT was extruded into 1.75 mm diameter filament with temperatures ranging from 170 °C to 210 °C. Profile temperature was set from the feeding zone to the nozzle. Then, the filament was pulled by Filament Maker-Composer 350 to obtain the required diameter. After exiting the extrusion apparatus, the filament was cooled to room temperature using the fans of the extruder. The standard deviation of the filament diameter was 5 μm. Three different types of filaments were prepared, and their abbreviations are presented in Table 2.

#### Design and Fabrication of 3D-Printed Specimens

Once the filament was fabricated, the PLA/MMT specimens for DMA, XRD, Water Contact Angle (WCA), and compression testing were printed using an XYZ da Vinci SUPER 3D printer (New Kinpo Group, New Taipei City, Taiwan). All of the specimen geometries were designed utilizing SolidWorks™ and converted into a stereolithography (STL) file format. The detailed printing parameters are summarized in Table 3. The specimens were printed with two outer layers (shell) with a concentric pattern, in rectilinear 45°/0° angle infill, with a 0.2 mm layer height and 100% infill percentage. The dimensions of the 3D-printed specimens for DMA were 40 mm × 6 mm × 2 mm, and their geometry was rectangular. Moreover, the specimens for contact angle tests were rectangular with dimensions of 30 mm × 10 mm × 3 mm. For XRD analysis, cylindrical specimens of 20 mm diameter and 2 mm height were manufactured. Additionally, cylindrical specimens of 12.5 mm diameter and 25 mm height were fabricated for compression testing. A support brim was employed for all the cylindrical specimens to improve contact with the building plate during 3D printing and was removed by the end of the printing process. For all specimens, slicing was performed using XYZprint, and finally, for the 3D process, a G-code was generated.

An XYZ da Vinci SUPER 3D printer with stainless steel nozzle of 0.4 mm diameter and a tempered glass building plate was used to manufacture the 3D-printed specimens for further study. The material was deposited layer by layer on a building plate by the extruder. Before setting the extruder temperature for all specimens, three different extruder temperatures (205 °C, 210 °C, and 215 °C) were preliminary evaluated for each filament to determine the ideal extruder temperature for 3D printing using a cylindrical plate design with a diameter of 20 mm and a height of 3 mm. For all PLA/MMT filaments, the best 3D printing quality performance was observed at 210 °C.

PLA/MMT filaments with 1, 2, and 4% (wt.) MMT content were fed into an XYZ da Vinci SUPER 3D printer, and the specimens were produced. No features of the 3D CAD design were smaller than the resolution limit of the FFF-printer of 0.4 mm. Before 3D printing, a paper sheet tape was applied on the surface of the glass building plate for better stability of the specimens during the 3D printing process.

### 2.3. Physicochemical Characterization

#### 2.3.1. Gel Permeation Chromatography

The molecular weight of the PLA/MMT nanocomposite filaments was investigated utilizing Gel Permeation Chromatography (GPC) with a Waters Alliance 2690 high-performance liquid chromatographic pump, Waters Ultrastyragel (Milford, MA, USA) with columns HR-1, HR-3, HR-4E, HR-4, and HR-4E, and a Waters Refractive Index Detector 2414 detector. Ten polystyrene (PS) standards with molecular weights ranging from 2500 to 900,000 g/mol were used for the calibration. The injection volume was 150 μL with a flow of 1 mL/min at a temperature of 40 °C, and the concentration of the prepared solutions was 10 mg/mL.

#### 2.3.2. Melt Flow Index (MFI)

According to the ASTM standard D 1238-04 and ISO standard 1133 (load 2.16 kg), the MFΙ of the PLA/MMT nanocomposite filament melts were measured at 210 °C with a CEAST’s Melt Flow Quick Index meter (CEAST, Turin, Italy).

#### 2.3.3. Scanning Electron Microscopy (SEM)

The top surfaces of the 3D-printed nanocomposites PLA/MMT were observed using a scanning electron microscope (Phenom ProX, ThermoFisher Scientific, Waltham, MA, USA). Firstly, an aluminum stub (placed in a charge reduction holder) was used to attach the samples utilizing double adhesive conductive carbon tabs (TED Pella, Redding, CA, USA) to secure them. Then, a carbon coating was applied on the surface of the samples to increase electrical conductivity, and the samples were scanned at an accelerating voltage of 15 kV. The samples were coated with carbon black, utilizing a sputter coater (SC 7620 model, Quorum Technologies, East Sussex, UK) for 90 s, 18 mA. Energy-Dispersive X-ray Spectroscopy (EDX) was performed on the surface of the specimens. The accelerating voltage was 15 kV.

#### 2.3.4. Fourier Transform Infrared Spectroscopy (FTIR)

FTIR spectra of the samples were recorded with an FTIR-2000 (Perkin Elmer, Waltham, MA, USA) spectrometer. The spectra were recorded in the range of 400 to 4000 cm^−1^ at a resolution of 4 cm^−1^ (a total of 16 co-added scans).

#### 2.3.5. X-ray Diffraction Analysis (XRD)

The X-ray diffraction patterns of the pure MMT and the PLA/MMT nanocomposites were obtained utilizing a MiniFlex II XRD system (Rigaku Co., Tokyo, Japan) with Cu Ka radiation (λ = 0.154 nm). The samples were measured in steps of 0. 05° across a 2θ range of 3 to 60° with a scanning speed of 1.5 deg/min. Using Bragg’s law, the interlayer distance of MMT was calculated at d = 2.45 nm.

#### 2.3.6. Thermal Analysis

A Perkin Elmer Pyris 6 DSC apparatus (Waltham, MA, USA), calibrated with indium and zinc standards, was used to conduct the Differential Scanning Calorimetry (DSC) measurements. Samples of 10 ± 2 mg were placed in sealed aluminum pans and heated up from 20 to 200 °C with a heating rate of 20 °C/min (N_2_, flow rate 50 mL/min). The degree of crystallinity was calculated using the following equation:

This is example 1 of an equation:

(1) $$Xc\left(\%\right)=\frac{\Delta Hm-\Delta Hcc}{\Delta H_{m}^{0}\times \left(1-\frac{additive\;wt\%}{100}\right)}\times 100\;$$

where Xc is the % crystallinity, ΔHm is the melting enthalpy, ΔHcc is the cold crystallization enthalpy, and Δ*H*^0^*_m_* is the melting enthalpy of 100% crystalline PLA. To examine the effect of the MMT concentration on the thermal stability of PLA, a thermogravimetric analysis (TGA) of all of the prepared nanocomposite filaments was carried out by a SETARAM SETSYS TG-DTA 16/18 instrument (Setaram, Lyon, France). The fabricated filaments’ mass loss and their first derivative curves were obtained. Using an empty alumina crucible as a reference, the samples (2 ± 0.2 mg) were placed in alumina crucibles. The samples were heated in an 8.3 × 10^−7^ m^3^ s^−1^ flow of N_2_ at a heating rate of 20 °C min^−1^ from room temperature to 600 °C.

#### 2.3.7. Water Contact Angle (WCA)

The apparent contact angle of the water was studied using a water contact angle tester (Ossila Contact Angle Goniometer L2004A1) to measure the surface wettability of the nanocomposites. The sessile drop method was used to analyze the WCA of the samples. Then, 25 μL of distilled water was added dropwise onto the top surface of the 3D-printed plates (*n* = 3). Images were captured within 20 s with a high-resolution camera and processed using the Ossila Contact Angle Software 4.0.3.1 (Ossila Ltd, Sheffield, UK).

### 2.4. Mechanical Characterization

#### 2.4.1. Nanoindentation Testing Supported by FEA

The nanomechanical properties of the PLA and PLA/MMT filaments were investigated using nanoindentation. A dynamic ultra-microhardness tester DUH-211 (Shimadzu Co., Kyoto, Japan) utilizing a 100 nm radius triangular pyramid indenter tip (Berkovich-type indenter) was used to determine the mechanical performance of PLA/MMT nanocomposites. During nanoindentation testing, a controlled load (P) with a peak load of 30 mN was applied through a diamond tip on the surface of the nanocomposite filaments and was held for 3 s. As a function of load, the indentation depth is recorded. The indenter was then unloaded, resulting in a load of zero. During the creep time, the maximum indentation load is applied to the indenter. The average value of ten measurements was used to calculate the modulus and hardness. A finite element analysis (FEA) process has been developed in order to fit the nanoindentation test curves and extract the stress–strain behavior of the PLA and PLA/MMT specimens. The interface between the indenter and the surface of the PLA/MMT nanocomposites was simulated with contact elements and assumed frictionless. The nanoindentation experiments have been computationally generated considering the simulation of the loading stage of the indenter penetrating into the surface of PLA/MMT specimens. Other works [35,36,37] have shown that kinematic hardening leads to a rapid convergence in the corresponding FEA calculations, so this method was utilized in the developed curve-fitting procedure.

#### 2.4.2. Compression Testing of Solid Specimens

To evaluate the mechanical behavior of the PLA/MMT nanocomposites, compression strength tests were performed using a universal testing system M500-50AT (Testometric, Rochdale, UK). For the compression testing, cylindrical structures with 100% infill and 12.5 mm diameter as well as 25 mm height were manufactured using FFF technology. The tests were carried out with a 50 kN load on the cell. For each different concentration of MMT in PLA, at least three specimens were tested, and the results were averaged to obtain the mean values of the Ei compression modulus and the ultimate compression stress of the PLA/MMT 3D-printed specimens. The compression tests were performed at room temperature (23 °C).

#### 2.4.3. Dynamic Mechanical Analysis (DMA)

The dynamic mechanical test was performed using a Diamond DMA Q8 (Perkin Elmer, MA, USA). The 3D-printed specimen’s dimensions were 40 mm × 6 mm × 2 mm, and their geometry was rectangular. At least three samples were 3D printed for each nanocomposite. They were measured in a 3-point bending mode with a frequency of 1 Hz oscillation. The experiments were carried out at a temperature ranging from 30 °C to 90 °C, and the heating rate was 3 °C min^−1^. The storage modulus (E′), loss modulus (E″), and loss factor (tan δ) were recorded as a function of temperature.

### 2.5. Statistical Analysis

The MFI, filament diameter, contact angle, and mechanical properties measurements were performed in triplicate (unless otherwise mentioned), and the results were expressed as mean ± standard deviation (SD). Unless otherwise stated, a one-way ANOVA with a post-hoc Tukey test was used. The software used was IBM SPSS Statistics 27 (IBM, New York, NY, USA). A *p*-value ≤ 0.05 was considered statistically significant.

## 3. Results and Discussion

### 3.1. Effect of MMT on Molecular Weight and Filament Preparation

The molecular weight of the filaments was measured with GPC. The M_n_, M_w_, M_p_, and PDI values are shown in Table 4. The M_n_ of PLA filament is 124,300 g/mol, and it gradually decreases with increasing MMT concentration, ranging from 113,400 g/mol to 75,900 g/mol. The M_n_ is higher than that of the PLA without 2 wt.% Joncryl before compounding (81,700 g/mol) for MMT content up to 2 wt.%. MMT has surface hydroxyl groups located at the edges of its layers that could react with the epoxides of the Joncryl chain extender [38,39], thus consuming the epoxides available for the chain extension of PLA and finally reducing the obtained M_n_.

The MFI is a parameter that gives insight into the FFF processing of polymers [40]. FFF requires a processing temperature above the melting point for the chains to move relative to each other, allowing the polymer to flow [41]. The MFI of the filaments was measured at 210 °C. PLA had an MFI of 0.37 ± 0.04, which significantly increased for all of the nanocomposite filaments, which means their fluidity was increased. This is in line with the decreasing trend of the M_n_ with increasing MMT concentration. Therefore, the increase in MFI can be attributed to the decrease in M_n_, but it has also been reported that the clays can act as internal lubricants for polymer melts, leading to decreased melt viscosities and subsequently increased MFIs [42].

The morphology, as well as the average diameter of the filaments, were investigated with the use of stereoscopic images (Figure S1), and the average value of 10 measurements is included in Table 4. According to the specifications of the 3D printer, the optimal diameter was 1.75 mm. While the filament of PLA had a smaller diameter, with an average value of 1.71 mm, the nanocomposites had slightly increased values. The filaments with MMT were of high quality and had slight diameter variation along their length. The PLA filament was transparent, whereases the PLA/MMT nanocomposite filaments were light yellow–brown.

**Figure 2:**
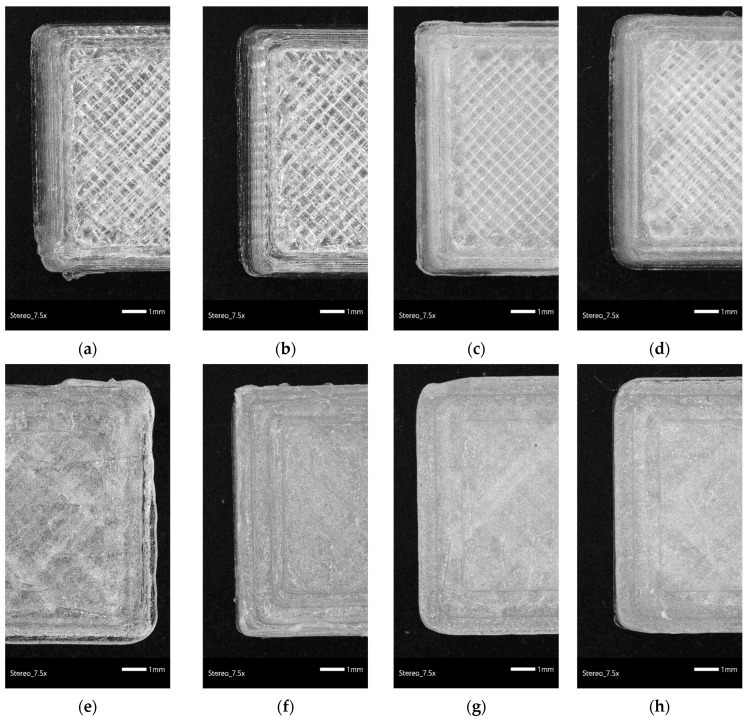
Stereoscope images of (**a**) PLA top view, (**b**) PLA/MMT1 top view, (**c**) PLA/MMT2 top view, (**d**) PLA/MMT4 top view and (**e**) PLA bottom view, (**f**) PLA/MMT1 bottom view, (**g**) PLA/MMT2 bottom view, (**h**) PLA/MMT4 bottom view of 3D-printed specimens through FFF technology.

The morphology of the 3D-printed specimens and the dimensional accuracy were examined with the help of stereoscopic images (Figure 2a–h). The 3D CAD model was designed as a square with dimensions of 10 mm × 10 mm × 3 mm. The dimensions (width and angle of the edges) of the 3D-printed specimens were measured at ten spots (both ends and the middle) using stereoscopic images of three identical specimens and compared with the original nominal values of the 3D CAD model to determine the 3D printing accuracy of the PLA and PLA/MMT nanocomposites. The average value of the width of the 3D-printed specimens of PLA, PLA/MMT1, PLA/MMT2, and PLA/MMT4 were measured to be 10.72 mm ± 0.09 mm, 10.24 mm ± 0.03 mm, 10.08 mm ± 0.05 mm, and 10.0 mm ± 0.03 mm, respectively. Furthermore, the average angles of the edges of the 3D-printed specimens were measured to be 89.30° ± 0.57, 89.65° ± 0.61, 89.71° ± 0.53, and 90.00° ± 0.34 for PLA, PLA/MMT1, PLA/MMT2, and PLA/MMT4, respectively. Furthermore, the thickness of the 3D-printed specimens was calculated using a calibrated electronic caliper at ten spots. The average values of the thickness of the 3D-printed specimens were 2.86 ± 0.02 mm, 3.05 ± 0.03 mm, 2.86 ± 0.05 mm, and 3.00 ± 0.02 mm for PLA, PLA/MMT1, PLA/MMT2, and PLA/MMT4, respectively. It is worth mentioning that, in terms of morphology, the use of MMT increased the 3D printing dimensional accuracy compared to PLA. Moreover, the presence of MMT promoted dimension and shape stability, better printing accuracy, and quality of the fabricated specimens.

**Figure 3:**
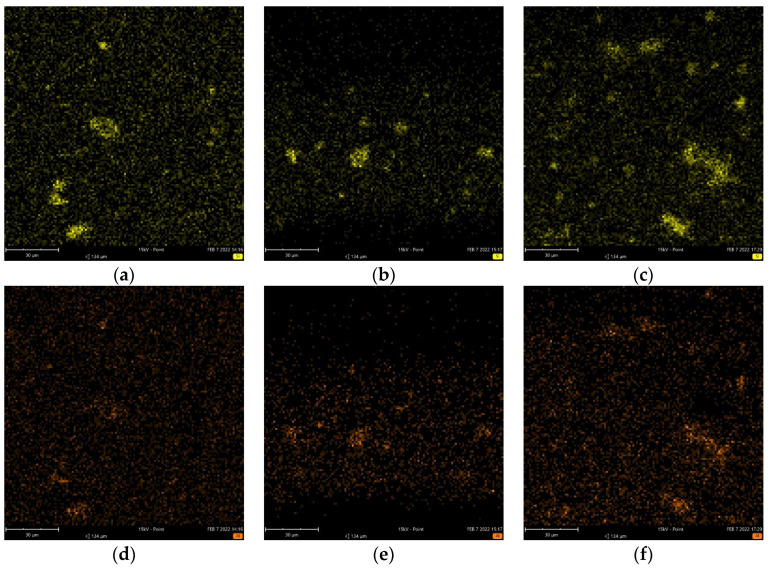
EDX color-mapping for (**a**) PLA/MMΤ1, (**b**) PLA/MMT2, (**c**) PLA/MMT4 showing the presence of Silicon (Si-yellow) and (**d**) PLA/MMΤ1, (**e**) PLA/MMT2, (**f**) PLA/MMT4 showing the presence of Aluminum (Al-orange).

Representative SEM images of the PLA/MMT nanocomposites are shown in Figure S2. EDX analysis was performed, and Figure 3a–f and Figure S3 present the element mapping images of PLA and PLA/MMT, while Table 5 summarizes energy spectrum analyses. The elements C, O, N, Si, Mg, and Al were detected through EDX in PLA and PLA/MMT. The presence of Si, Al, and Mg confirm the presence of MMT and also prove that MMT was successfully printed out through the 3D printer nozzle without clogging. The dispersion can be assessed from color-mapping images, where many homogeneously distributed color points of Si and Al can be noticed all over the surface of 3D-printed nanocomposite specimens, which implies the uniform distribution of the nanofiller in the PLA matrix. The Si wt.% content increases with increasing MMT content as expected, and it is about 1.27%, 1.71%, and 3.12% for PLA/MMT1, PLA/MMT2, and PLA/MMT4, respectively. Moreover, a slight increase in the Al content in PLA/MMT4 can be noticed. Some aggregates were formed due to insufficient dispersion from the use of the homogenizer and the process described in Section 2.2. The color maps show such a concentrated concentration of Si and Al, but overall, the particles are distributed in the whole field of vision.

Although the nanoparticles tend to agglomerate, from the SEM and EDX analyses, it can be observed that the presence of Joncryl and the preparation of MMT before mixing led to the good dispersion of MMT nanoparticles in the PLA polymer matrix. SEM micrographs and the EDX results show the good incorporation of MMT into the PLA nanocomposite filaments on the morphology of the developed 3D-printed specimens. It has been reported that the addition of chain extenders to PLA decreased the agglomeration of particles and distributed the particles better due to the increase in the viscosity of PLA by chain extension or branching [23]. In addition to the chain extender, the pretreatment of the filler with ultrasonication, turrax, and lyophilization could have improved the distribution of MMT nanoparticles in the polymer, as it ensures no big aggregates are added to the polymer during filament fabrication.

### 3.2. Structural Properties

**Figure 4:**
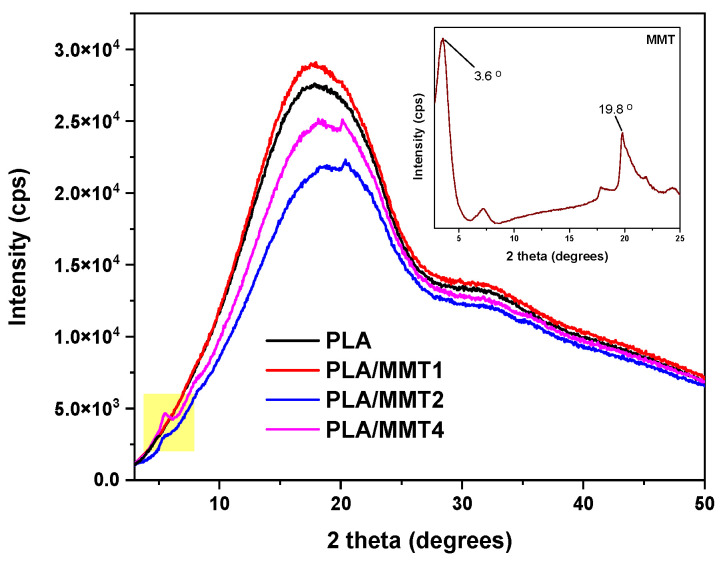
X-ray diffraction patterns of PLA, PLA/MMT and MMT.

Figure 4 illustrates the XRD graphs of the pure PLA and PLA/MMT nanocomposites. The PLA was amorphous, as witnessed by the lack of sharp diffraction peaks. The peak of the (001) reflection MMT at 3.6° (Figure 4 inset) corresponds to the basal spacing of d_001_ = 2.45 nm of MMT. At 7.2°, the secondary (002) plane reflection is visible, with d_002_ = 1.23 nm, which suggests that a low content of MMT layers without organic modifier insertion is present [43].

When MMT is mixed with polymers, the polymer chains tend to intercalate between the layers of the clay resulting in an increase in the basal spacing, which is reflected as a shift in the diffraction peak of MMT towards smaller angles [44,45]. In the diffractogram of the nanocomposite PLA/MMT1, no peaks associated with the (001) plane of MMT are visible. This could be due to either the small content of MMT, which causes it to be undetectable, or due to the exfoliation of the MMT layers, or the position of the peak at 2θ < 3°, which is out of the range of the instrument. On the other hand, the nanocomposites PLA/MMT2 and PLA/MMT4 show additional peaks at 2θ = 5.5° (d_002_ = 1.61 nm) and 2θ = 20.2°, which correspond to the secondary reflection of MMT and the (203) plane of crystalline PLA or the (003) plane of MMT, respectively. The shifting of the d_002_ peak to smaller angles is evidence of the intercalation of PLA within the MMT gallery space [46,47]. Because of the overlap of the peak associated with the (203) plane of PLA that appears at around 20° and the peak of MMT at 19.8°, it is difficult to determine whether it is attributed to the PLA crystals or MMT.

**Figure 5:**
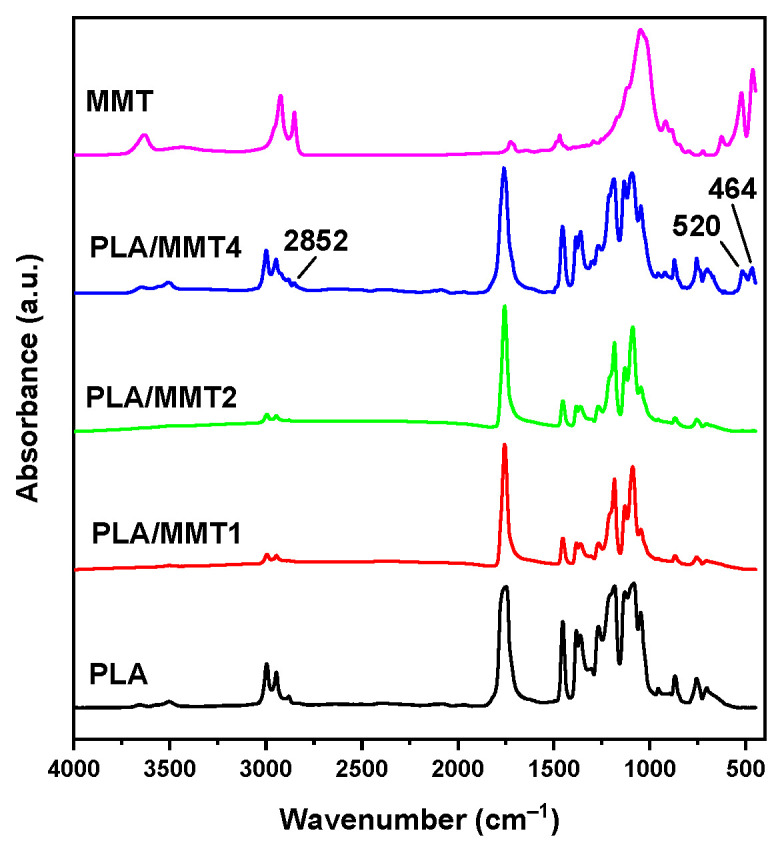
FTIR spectra of PLA, its nanocomposites with MMT, and neat MMT.

The FTIR spectra of PLA, MMT, and their nanocomposite filaments are shown in Figure 5. MMT has strong absorption bands at 3633 cm^−1^ 2923 cm^−1^, 2851 cm^−1^, 1048 cm^−1^, and 463 cm^−1^. These bands are assigned to O–H stretching, C–H asymmetric stretching, C–H symmetric stretching, Si–O–Si stretching, and Al–O–Si bending, respectively [32]. PLA shows the characteristic absorption bands at 3700–3500 cm^−1^ assigned to O–H bending, at ~2900 cm^−1^ of C–H stretching, at 1749 cm^−1^ caused by the C=O stretching of polyesters, at 1456 cm^−1^ of –CH_3_ asymmetric bending, and C–O–C stretching at 1183 cm^−1^, 1136 cm^−1^, and 1084 cm^−1^. The bending vibrations of the C–H bending of the CH–OH end group appear at 1044 cm^−1^. The nanocomposites PLA/MMT1 and PLA/MMT2 do not show any new bands, likely because of the small MMT content. On the other hand, the nanocomposite PLA/MMT4 has three small but additional detectable bands, at 2852, 520, and 464 cm^−1^, which were assigned to the vibrations of the groups of MMT.

### 3.3. Thermal Properties

The thermal properties of the developed PLA/MMT nanocomposites were investigated by DSC and TGA and were performed on the extruded filaments. The recorded DSC thermograms are presented in Figure S4. The characteristic thermal transitions, including the glass transition temperature (T_g_), the cold crystallization temperature (T_cc_), and the melting point (T_m_), as well as the % crystallinity of the materials, are summarized in Table 6. The endothermic peak right after the glass transition is due to enthalpy relaxation [48]. High-molecular-weight PLA is unable to crystallize during cooling [49,50]. Indeed, all of the filaments were amorphous after cooling the filament to room temperature, despite the reduction in the molecular weight of the composites and the presence of MMT, which is expected to act as a heterogenous nucleation agent because Joncryl introduces branched points into the macromolecular chains, which suppress crystallization, in addition to the large molecular weight of the nanocomposites [51]. Additionally, no crystallization was detected during cooling from the melt at a rate of 10 °C. The T_cc_ and the melting peaks, which were the result of cold crystallization during heating, as well as the T_g_, shifted towards higher temperatures after the addition of MMT. This can be attributed to the reduced chain mobility because of the chain extension and the presence of the nanofillers. Cold crystallization of PLA/MMT2 and PLA/MMT4 in particular not only starts in lower temperatures, but the ΔH_cc_ (area of the peak) increases, meaning that cold crystallization is more pronounced. This could be due to the decreased molecular weight of the two nanocomposites and the action of MMT as a heterogeneous nucleation agent [52,53]; when the MMT content is larger than 1 wt.%, this effect seems to dominate over the suppression of crystallization because of the branching caused by the chain extender.

**Figure 6:**
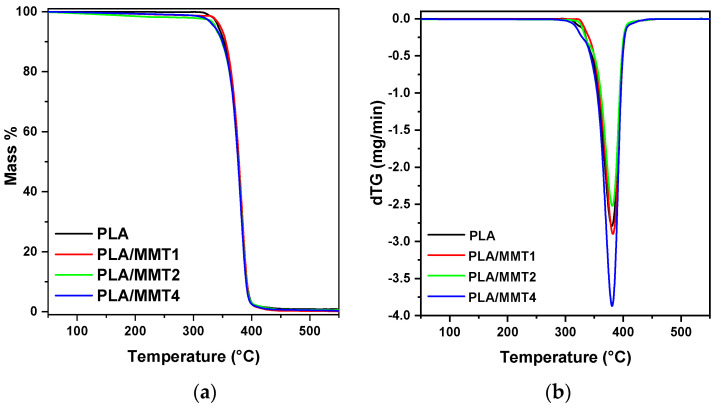
(**a**) TGA and (**b**) dTG curves of the filaments.

The thermal stability of the PLA and PLA/MMT nanocomposite filaments was measured using thermogravimetric analysis (TGA). The mass loss and DTG curves of PLA and its nanocomposite filaments are shown in Figure 6a,b, and the thermal degradation characteristics are in Table 7. According to the literature, the onset decomposition temperature of MMT from TGA is 198 °C, and the maximum mass loss rate temperatures from DTGA are 336 °C and 451 °C [32]. Both the T_p_ and the extrapolated T_o_ of the PLA filament increase in the presence of MMT, with the increase being more prominent in the case of T_o_, demonstrating the positive effect of MMT on the thermal stability of PLA. The beneficial effect of nanoclays on the thermal stability of polymers can be attributed to the physical barrier that the layers create for heat transfer and the decomposition products; thus, delaying their volatilization [53,54,55,56,57], which prevailed against the reduction in molecular weight that might have otherwise reduced thermal stability. The deceleration of the degradation due to the clays is also associated with the good dispersion of the exfoliated layers and intercalated stacks, creating a more tortuous path that the volatile degradation products have to cross; thus, hindering their diffusion [24,58,59].

### 3.4. Water Contact Angle

The hydrophilicity of the PLA and PLA/MMT nanocomposites was examined using water contact angle measurements. In Figure S5, the water contact angle values of the 3D-printed specimens of PLA and PLA/MMT nanocomposites are presented. PLA had a contact angle of ~60°. The addition of MMT to PLA caused an increasing trend of the contact angle from 60° up to 70° due to the hydrophobic nature of the modifier of MMT, which contains tallow, i.e., triglycerides with long aliphatic chains, as well as the increase in surface roughness that the nanoclays can impart [60]. However, the one-way ANOVA test showed that there is no major difference between the contact angle values (*p* > 0.05). The increasing trend agrees with previous studies [61], and all of the nanocomposites remained hydrophilic since their water contact angle was smaller than 90°.

### 3.5. Mechanical Properties

#### 3.5.1. Mechanical Characterization through FEA-Assisted Nanoindentation and Compression Testing

**Figure 7:**
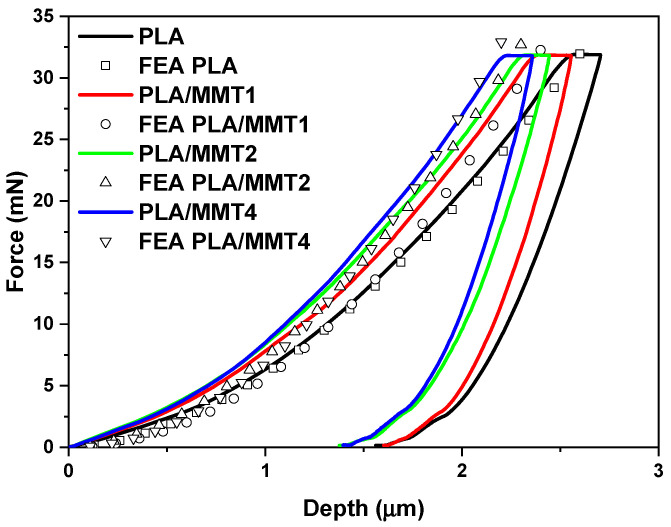
Comparison of load–depth nanoindentation curves of PLA and PLA/MMT nanocomposites.

The mechanical behavior of the PLA/MMT nanocomposites was evaluated by nanoindentation and compression testing. Nanoindentation testing was used to determine the hardness and modulus of elasticity of the PLA/MMT nanocomposites that were suitable for 3D printing. The nanoindentation tests were performed on the extruded filaments to evaluate the influence of the addition of MMT into the PLA matrix. The results were also used as an input for an FE analysis of the mechanical behavior of the nanocomposite materials under study. The improved mechanical properties of PLA after the incorporation of MMT are noticeable through the results of the nanoindentation tests. In Figure 7, the representative indentation load–depth curves are illustrated as measured from the nanoindentation and compared to the PLA filament as a control sample, according to our previous work [29]. The maximum indentation depths at peak load for the PLA/MMT nanocomposites varied approximately between 2.21 and 2.56 μm. The range of the nanoindentation depth was 2.55 to 2.70 μm for PLA, 2.38 to 2.56 μm for PLA/MMT1, 2.31 to 2.44 μm for the PLA/MMT2 filament, and 2.21 to 2.35 μm for PLA/MMT4. According to the results of the nanoindentation testing, the stiffening effect that occurred with the introduction of MMT into PLA can be clearly noticed despite the smaller M_n_ of the nanocomposites.

For the FE analysis, an initial value was assumed for the first tangent modulus of the sample’s stress–strain curve. This value is related to the elastic modulus that has been extracted by the nanoindentation experiments. The measured indentation depth is applied in steps to the FE model (on the indenter), and then the force reaction is then computed and compared to the measured value. The maximum indentation depth values applied in the computational models ranged from 2.70 μm to 2.56 μm for the neat PLA and PLA/MMT, respectively. The FEA force–depth data should fit the experimental nanoindentation curve; else, the value of the tangent modulus has to be computed again. For the cases where the solutions returned a computational force matching the measured force, the value of the tangent modulus was considered to be accepted, and the next couple of values of force and depth were applied to the model. The following calculation steps started with the previous indentation depth value, considering the already existing stress status and the previously obtained tangent modulus. This process is repeated until the last couple of load–depth values converge and the loop ends. The computationally generated force–depth curves are presented in Figure 7. The results show a good correlation between the measured indentation tests and the computational data of the PLA and PLA/MMT specimens in any case. At least 20 steps of simulation were considered sufficient to achieve converged FEA solutions and proceed to a satisfactory curve fitting of the nanoindentation curves. The potential to calculate the stress–strain curves of polymers based on force–depth nanoindentation testing under varied conditions allows for the estimation of the materials’ constitutive laws.

**Figure 8:**
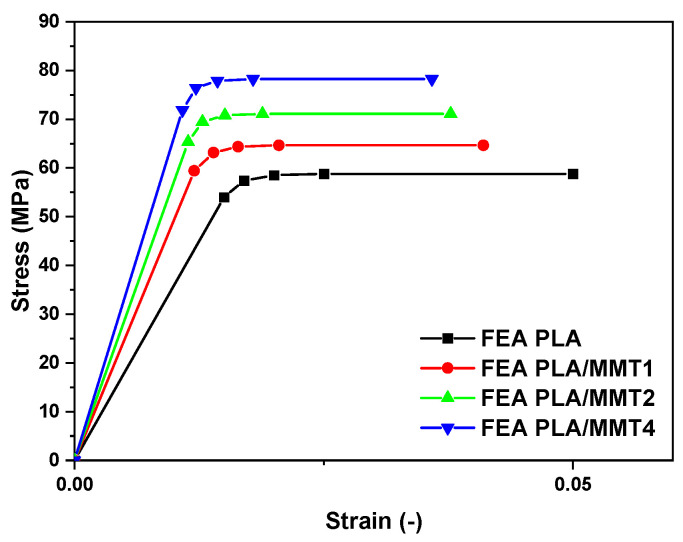
Curve fitting between the experimental and the FEA generated stress–strain curves of the PLA and PLA/MMT.

The stress–strain response was assessed by the optimal curve fitting force–depth results that matched the nanoindentation experimental data. The FEA results revealed acceptable values of the elasticity moduli for both the neat PLA and the PLA/MMT nanocomposites specimens. Table 8 shows this convergence between FEA and the experimental results for all of the PLA/MMT samples. Figure 8 illustrates the FEM-extracted stress–strain laws of the PLA/MMT specimens. The results show a significant increase in strength for the MMT-reinforced PLA specimens. The PLA/revealed higher elasticity moduli and strength compared to the other nanocomposites. Considering these results, it can be concluded that the MMT inclusions affected the specimen’s mechanical properties and their overall stress–strain behavior. Furthermore, the experimental nanoindentation technique assisted by FEA has proven to be a very successful method for determining the mechanical behavior of PLA and PLA/MMT nanocomposites.

**Figure 9:**
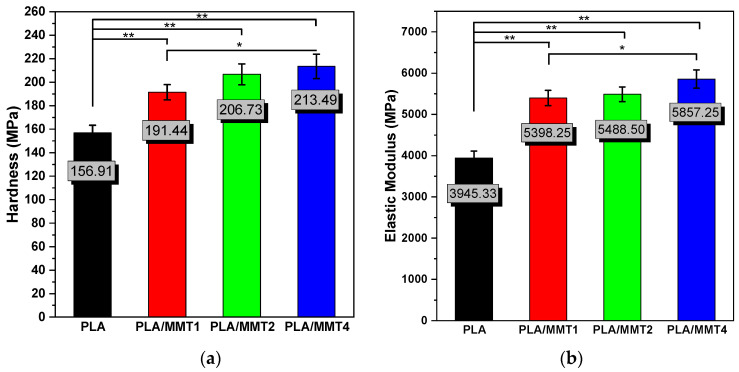
Effect of MMT content on the (**a**) hardness and (**b**) elastic modulus of PLA/MMT filaments. * *p* ≤ 0.05, ** *p* ≤ 0.01.

The calculation method to determine the elastic modulus and hardness of the nanocomposite materials used for the 3D-printed specimens was based on Oliver and Pharr [62] and previous work [35,36,63,64,65]. In Figure 9a,b, the calculated nanomechanical properties of the PLA/MMT nanocomposite filaments are summarized as hardness and elastic modulus measured by nanoindentation and as a function of MMT concentration. The average values of the hardness and elastic modulus of the PLA filaments were presented based on previous work [29]. The hardness and elastic modulus of PLA were measured to be 156.91 MPa and 3945.33 MPa, respectively. The hardness and elastic modulus significantly increased with the addition of 4 wt.% MMT.

**Figure 10:**
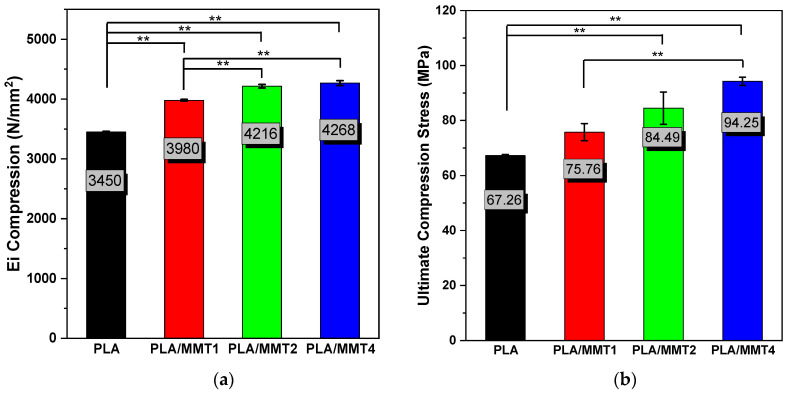
Effect of MMT content on the (**a**) *E*_i_ Compression Modulus and (**b**) Ultimate Compression Stress of PLA/MMT 3D-printed specimens. ** *p* ≤ 0.01.

Compression tests were conducted to investigate the mechanical response of the PLA/MMT nanocomposites with three different MMT loadings. Figure 10a,b shows the compression results of the PLA/MMT 3D-printed solid specimens compared to the PLA values. The average and standard deviation of compressive modulus were equal to 3450 ± 9.79 MPa, 3980 ± 14.50 MPa, 4216 ± 29.46 MPa, and 4268 ± 39.80 MPa for PLA, PLA/MMT1, PLA/MMT2, and PLA/MMT4, respectively. Furthermore, the average ultimate compression stress of PLA, PLA/MMT1, PLA/MMT2, and PLA/MMT4 were 67.26 ± 0.39 MPa, 75.76 ± 3.09 Mpa, 84.49 ± 5.88 Mpa, and 94.25 ± 1.50 MPa. Comparing the results of compression tests, it can be noticed that PLA shows poor mechanical behavior compared to nanocomposites PLA/MMT. The mechanical behavior significantly improved with the addition of MMT, and the difference is remarkable, especially in the PLA/MMT4 nanocomposite. Regarding the mean values of the compression modulus and ultimate compression stress, the highest mechanical behavior is obtained at 4 wt.% MMT loading in the PLA matrix. These results agree with the mechanical properties of the PLA/MMT nanocomposites obtained by nanoindentation testing. In conclusion, it can be observed that PLA/MMT4 demonstrated optimal mechanical behavior through nanoindentation and compression tests.

#### 3.5.2. Dynamic Mechanical Analysis (DMA) of 3D-Printed Specimens

**Figure 11:**
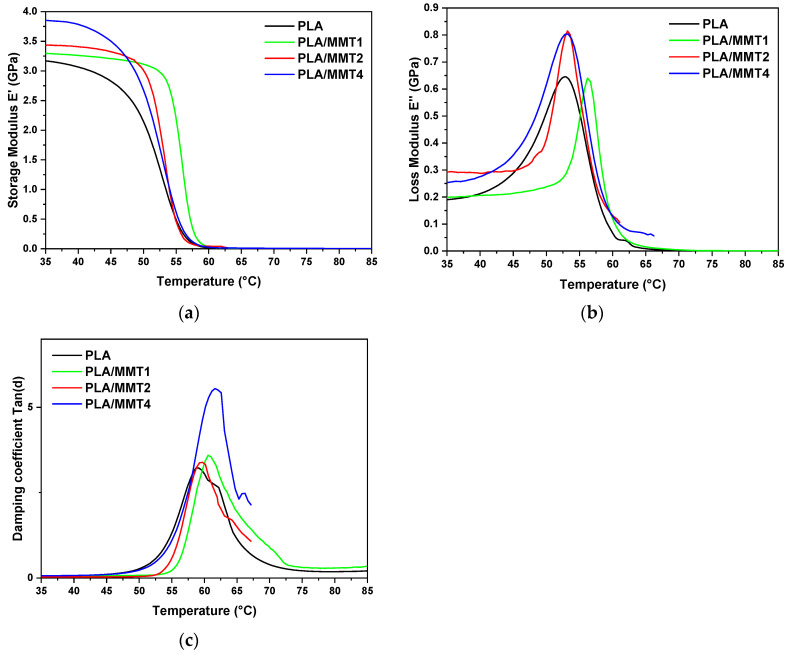
DMA plots of (**a**) Storage Modulus E′, (**b**) Loss Modulus E″ and (**c**) loss factor tan δ as a function of temperature for the 3D-printed specimens of PLA/MMT.

Rectangular specimens of each nanocomposite PLA/MMT were 3D printed and evaluated with dynamic mechanical analysis to assess their viscoelastic behavior and effect on 3D printing. The values of the dynamic storage modulus (E′), dynamic loss modulus (E″), and tangent delta (tan δ) were investigated, and the experimental results are presented in Figure 11a–c.

The addition of MMT to PLA imparts a higher storage (E′) and loss modulus (E″) than PLA, as shown in Figure 11. In Figure 11a, at 35 °C, a general increase in E’ with increasing nanofiller content is observed. Moreover, a remarkable difference in PLA/MMT4 in the glassy state can be noticed. The effect of the addition of MMT into the PLA matrix on the loss modulus can be observed in Figure 11b. It is clear that the PLA/MMT4 specimen demonstrated the highest loss modulus, while the addition of MMT to PLA progressively increased the loss modulus. In Figure 11c, the typical tan δ curves for determining the glass transition of the 3D-printed PLA/MMT nanocomposites are presented. Generally, the tan δ peak represents the ratio of loss to the storage modulus of the material per cycle, and it can be used to evaluate the T_g_ of the polymers. The T_g_ increased slightly as the nanoclay content increased from 58.7 °C for PLA to 61.6 °C for the nanocomposite PLA/MMT4. The increasing trend of T_g_ seems to be due to the enhanced interaction between the nanoclay and polymer matrix, which restricts chain segmental motion [66]. The comparative values of E’ for PLA/MMT at 35 °C and 55 °C and T_gtanδ_ are presented in Table 9. The difference in the T_g_ values that were achieved from DMA analysis compared with the DSC values could be a result of the 3D printing process. Furthermore, it is known that the dynamic mechanical characteristics seem to be more sensitive to local segmental motion than the thermal characteristics determined by DSC analysis [66]. Additionally, it can be observed that tan δ peak height increased slightly as the MMT concentration increased, with a higher peak of 5.01 for PLA/MMT4. Using two different codes of PLA (4032D and 2003D) and Cloisite 30B^®^ as a nanoclay filler, Coppola et al. [24] reported lower peak height and a smaller area than the neat PLA matrix for nanocomposites with 4 wt.% MMT due to the confinement effect by the presence of nanoclay, as well as the increase in the elastic component more than viscous and the decrease in polymer-chain mobility. Furthermore, the tan δ area is approximately the same for PLA and PLA/MMT1, but a decrease in PLA/MMT1 can be noticed and a slight increase from 28.5 °C for PLA to 32.8 °C for PLA/MMT4. In conclusion, the highest values of E’ at a temperature of 35 °C, as well as tan δ peak height and tan δ peak area, were observed for the PLA/MMT nanocomposite with 4 wt.% MMT. This could indicate that there no significant occurrence of confinement phenomena, which could be explained by the presence of Joncryl in the PLA/MMT nanocomposite filaments. The pretreatment of MMT prior to its mixing could be another reason that there is no significant agglomeration of MMT at the highest value of ΜΜΤ content.

## 4. Conclusions

In this study, PLA/MMT filaments and 3D-printed constructs with different MMT contents were successfully prepared using FFF technology, giving an insight into the fabrication process, printability, and melt flow index of the nanocomposite filaments. The 3D-printed PLA specimens with three different contents of MMT (1 wt.%, 2 wt.%, and 4 wt.%) were comprehensively analyzed in terms of their physicochemical and mechanical properties.

According to the SEM and EDX analyses, even after 3D printing, the MMT nanoparticles were well dispersed within PLA. These findings indicate that both the presence of Joncryl and the freezing/thawing-ultrasonic exfoliation MMT pretreatment improved the distribution of the MMT nanoparticles in the PLA matrix. The addition of MMT improved the 3D filament quality of PLA since the nanocomposite PLA/MMT filaments presented excellent printability through FFF technology. In terms of morphology, MMT improved the printing accuracy compared to PLA. Moreover, the 3D-printed PLA/MMT specimens demonstrated enhanced mechanical properties. According to the findings of mechanical characterization by nanoindentation testing, the addition of MMT to PLA increased the hardness as the MMT content increased. PLA/MMT nanocomposite filaments showed better mechanical properties when using 4 wt.% concentration of MMT.

Moreover, one of the novelties of this work is the direct extrusion of nanocomposite materials without using any process such as melt mixing or nanocomposite masterbatch fabrication. Furthermore, the presence of Joncryl and MMT enhanced the printability and the mechanical behavior of the PLA and PLA/MMT nanocomposite filaments; therefore, they could be excellent materials for 3D printing applications. Moreover, the pretreatment of MMT using the freezing/thawing-ultrasonic exfoliation method could be the reason behind the improved mechanical behavior and the lack of agglomeration at the highest MMT loading (4 wt.%), according to the tan δ peak analysis. The direct extrusion of low-cost biobased nanocomposites, such as PLA/MMT, simplifies the process and reduces the thermal and mechanical stress of the materials before extrusion. As a result, nanocomposite filaments with optimal mechanical and thermal properties suitable for 3D printing applications could be produced. At the same time, the fabrication time and the production cost are minimized, which is crucial for the potential use of such materials in any industrial field. As 3D printing allows the fabrication of customizable designs that have geometrical complexity, the successful 3D printing of PLA/MMT nanocomposites with excellent mechanical and thermal properties will help to create cost-efficient, sustainable alternatives for 3D printing complex geometries and porous constructs, such as scaffolds, and would be a promising candidate for biomedical applications.

## Figures and Tables

**Table 1:** Properties of PLA 3052D provided by the supplier.

Properties	Value
Specific Gravity	1.24
Crystalline Melt Temperature (°C)	145–160
Glass transition Temperature (°C)	55–60
Clarity	Transparent

**Table 2:** The composition and abbreviations of the fabricated filament.

Abbreviation	PLA Content (wt.%)	Joncryl Content (wt.%)	MMT Content (wt.%)
PLA	100	2	0
PLA/MMT1	97	data	1
PLA/MMT2	96	data	2
PLA/MMT4	94	data	4

**Table 3:** Printing parameters of FFF 3D printing for XRD, DMA, WCA, and compression specimens of PLA and PLA/MMT nanocomposites.

Properties	Value
Infill density	100%
Layer thickness of first layer	0.35 mm
Layer thickness	0.2 mm
Number of shells	2
Raster orientation	45°/0°
Print speed	30 mm/s
Extruder temperature	210 °C
Printing bed temperature	45 °C

**Table 4:** Molecular weight, PDI, MFI, and diameter of PLA/MMT filaments.

Sample	M_n_ (g/mol)	M_w_ (g/mol)	M_p_ (g/mol)	PDI	MFI (g/10 min)	Average Filament Diameter (mm)
PLA	124,300	210,900	172,100	1.60	0.37 ± 0.04	1.71 ± 0.01
PLA/MMT1	113,400	201,300	167,300	1.77	1.16 ± 0.16 **	1.76 ± 0.02 **
PLA/MMT2	107,700	194,700	154,200	1.80	1.23 ± 0.04 **	1.79 ± 0.01 **
PLA/MMT4	75,900	135,700	97,000	1.78	0.99 ± 0.12 **	1.78 ± 0.02 **

M_n_: number average molecular weight, M_w_: weight average molecular weight, M_p_: peak maximum molecular mass, PDI: polydispersity index. ** *p* ≤ 0.01 in comparison with neat PLA.

**Table 5:** Energy spectrum analysis of SEM results.

Element Symbol	Atomic Conc.			
	PLA	PLA/MMT1	PLA/MMT2	PLA/MMT4
C	91.05	83.15	82.99	81.57
O	8.95	6.42	6.68	6.77
N		7.32	7.10	6.5
Si		1.27	1.71	3.12
Al		0.93	0.92	1.37
Na		0.36	0.22	0.19
Mg		0.55	0.38	0.49

**Table 6:** Characteristic thermal transition temperatures of PLA and PLA/MMT filaments.

Sample	T_g_ (°C)	T_cc_ (°C)	T_m_ (°C)	ΔH_cc_ (J/g)	Crystallinity (%)
PLA	64.0	130.4	149.5	−2.6	0.2
PLA/MMT1	64.9	130.1	152.4	−5.7	0
PLA/MMT2	66.0	120.7	155.6	−6.5	0
PLA/MMT4	64.4	123.1	155.3	−15.2	0

**Table 7:** Thermal degradation characteristics of PLA neat, PLA, PLA/MMT filaments.

Sample	*T*_o_ (°C)	*T*_d,10%_ (°C)	*T*_p_ (°C)
PLA	359.6	352.3	380.5
PLA/MMT1	364.7	355.5	382.5
PLA/MMT2	364.4	348.8	381.1
PLA/MMT4	367.0	347.8	380.8

T_o_: extrapolated onset of degradation (the point of intersection of the starting-mass baseline and the tangent to the TGA curve at the point of maximum gradient), T_d,10%_: temperature that corresponds to 10% mass loss, T_p_: peak temperature of DTG where degradation occurs with the fastest rate.

**Table 8:** Comparison of elastic moduli of nanoindentation of PLA and PLA/MMT.

Sample	*E*_i_ Nanoindentation (N/mm^2^)	*E*_i_ Nanoindentation FEA (N/mm^2^)
PLA	3945.33 ± 134.74	3600
PLA/MMT1	5398.25 ± 184.80	4950
PLA/MMT2	5488.50 ± 177.31	5732
PLA/MMT4	5857.25 ± 222.11	6637

**Table 9:** DMA analysis results, including Dynamic modulus (E′), glass transition temperature (T_g_), and loss tangent (tan δ) of PLA and PLA/MMT 3D-printed specimens.

Sample	E′_35 °C_ (MPa)	E′_60 °C_ (MPa)	T_gtan__δ_ (°C)	(tan δ)_height_	(tan δ)_area_
PLA	3167.63	21.58	58.70	3.08	28.50
PLA/MMT1	3296.72	43.67	60.60	3.43	27.55
PLA/MMT2	3433.71	43.87	59.70	3.29	20.22
PLA/MMT4	3851.89	30.03	61.60	5.01	32.80

## Data Availability

Not applicable.

## Supplementary Materials

The following supporting information can be downloaded at: https://www.mdpi.com/article/10.3390/nano12152641/s1, Figure S1: Stereoscope images of PLA and PLA/MMT filaments through FFF technology, Figure S2: SEM microstructure of the top view of 3D-printed specimens (a) PLA, (b) PLA/MMT1 (c) PLA/MMT2 and (d) PLA/MMT4, Figure S3: EDX color-mapping for PLA (first column) presentation of signal for Carbon and Oxygen (C-green, O-blue, respectively), PLA/MMT1 (second column), PLA/MMT2 (third column), and PLA/MMT4 (fourth column) showing the presence of C, O, Nitrogen (N-magenta), and Magnesium (Mg-light blue) Energy spectrum analysis of SEM, Figure S4: DSC traces of neat PLA and PLA/MMT nanocomposites filaments during heating at 20 °C/min. Figure S5: The water contact angle of PLA and PLA/MMT nanocomposites.
