# Supplementary material for: Physicochemical Characterization and Finite Element Analysis-Assisted Mechanical Behavior of Polylactic Acid-Montmorillonite 3D Printed Nanocomposites
Source: Nanomaterials (Basel). 2022 Jul 31;12(15):2641. doi: 10.3390/nano12152641 (PMC9370662; doi:10.3390/nano12152641)
Supplement: Supplementary file 1 [file nanomaterials-12-02641-s001.zip › nanomaterials-1807265-supplementary.pdf]

nanomaterials

Supplementary Material

Physicochemical Characterization and Finite Element Analysis-Assisted Mechanical Behavior of Polylactic Acid-Montmorillonite 3D Printed Nanocomposites

Maria-Eirini Grigora [1], Zoe Terzopoulou [2,*], Konstantinos Tsongas [1], Dimitrios N. Bikiaris [2] and Dimitrios Tzetzis [1,*]

[1] Digital Manufacturing and Materials Characterization Laboratory, School of Science and Technology, International Hellenic University, Thessaloniki, 57001, Greece; megrigora@ihu.edu.gr (M.-E.G.); k.tsongas@ihu.edu.gr (K.T.)
[2] Laboratory of Polymer Chemistry and Technology, Department of Chemistry, Aristotle University of Thessaloniki, 54124 Thessaloniki, Greece; dbic@chem.auth.gr
* Correspondence: d.tzetzis@ihu.edu.gr (D.T.)

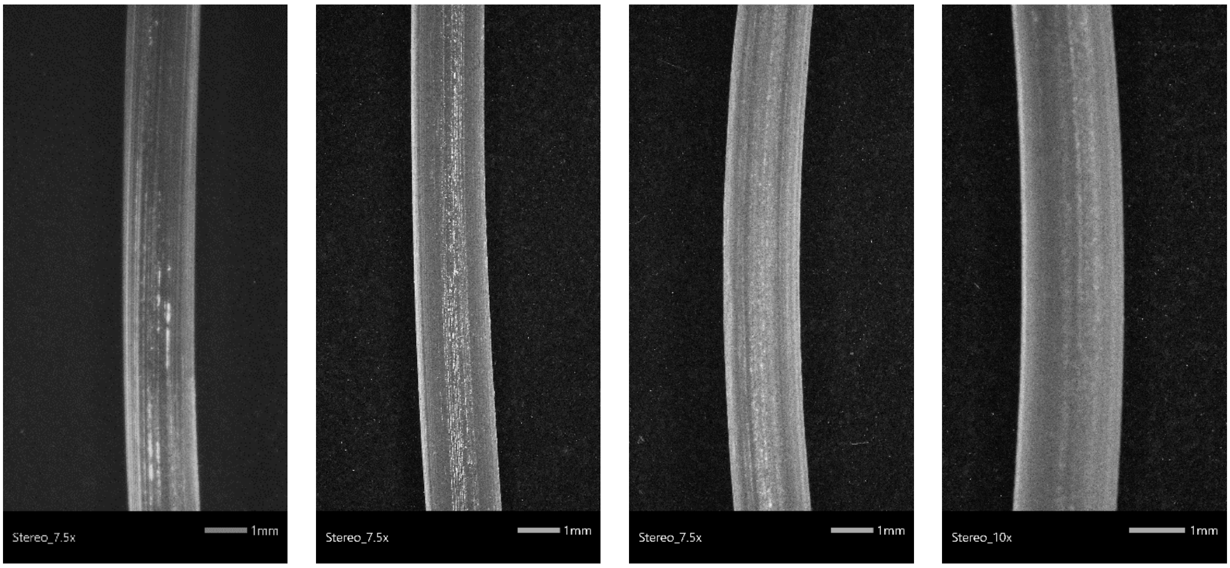

(**a**) (**b**) (**c**) (**d**)

Figure S1. Stereoscope images of (**a**) PLA, (**b**) PLA/MMT1, (**c**) PLA/MMT2 and (**d**) PLA/MMT4 filaments through FFF technology.

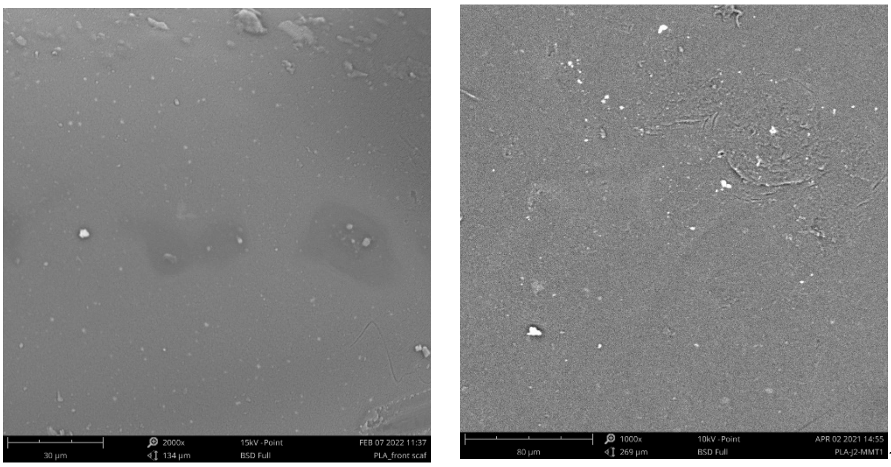

(**a**) (**b**)

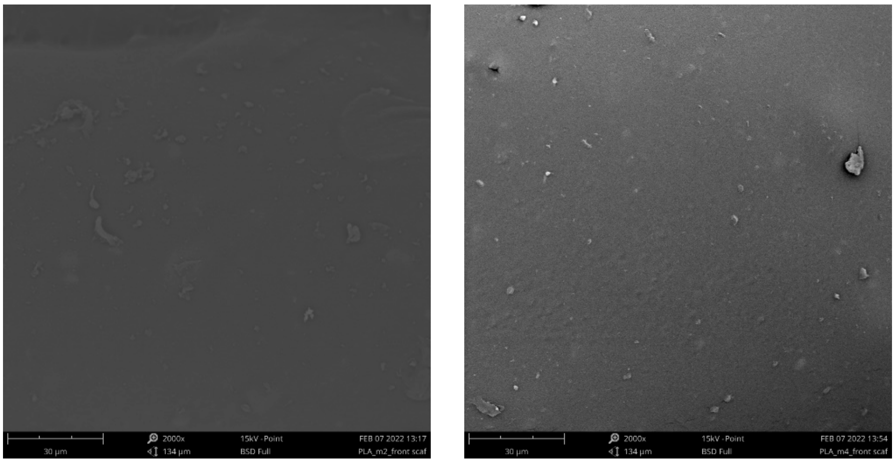

(**c**) (**d**)

Figure S2. SEM microstructure of the top view of 3D printed specimens (**a**) PLA, (**b**) PLA/MMT1 (**c**) PLA/MMT2 and (**d**) PLA/MMT4.

PLA **PLA/MMT1** **PLA/MMT2** **PLA/MMT4**

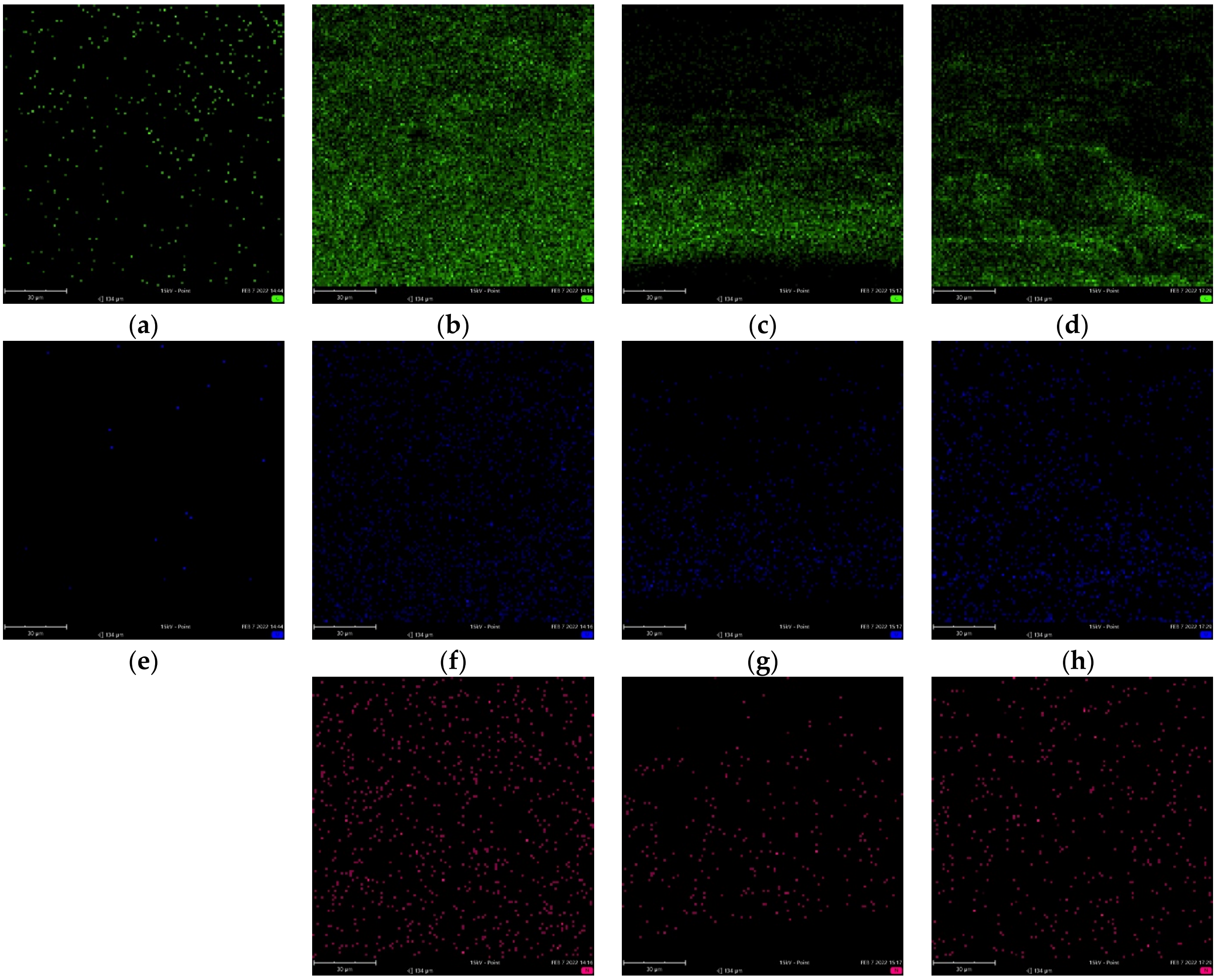

(**a**) (**b**) (**c**) (**d**)

(**e**) (**f**) (**g**) (**h**)

(**i**) (**j**) (**k**)

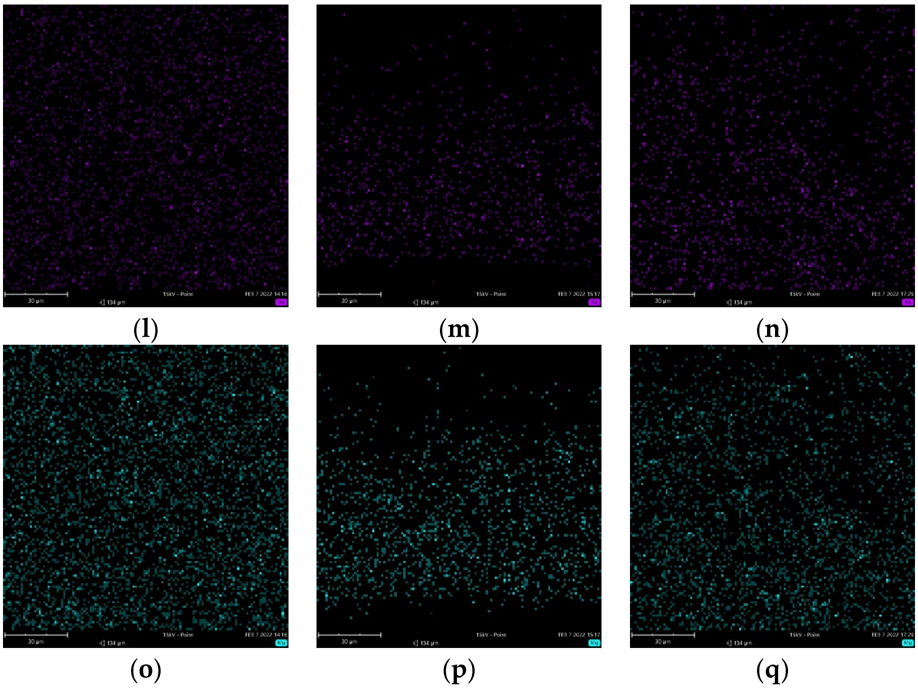

Figure S3. EDX color-mapping for PLA (first column) presentation of signal for Carbon (**a**–**d**) and Oxygen (**e**–**h**) (C-green, O-blue, respectively), PLA/MMT1 (second column), PLA/MMT2 (third column) and PLA/MMT4 (fourth column) showing the presence of C, O, Nitrogen (N-magenta (**i-k**)), Sodium (Na-purple (**l-n**)) and Magnesium (Mg-light blue (**o-q**)) Energy spectrum analysis of SEM.

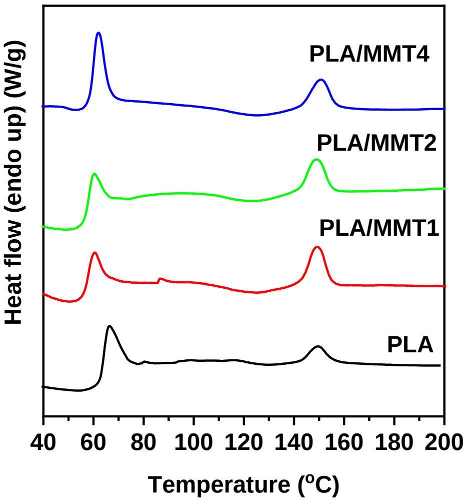

Figure S4. DSC traces of neat PLA and PLA/MMT nanocomposites filaments during heating with 20 °C/min.

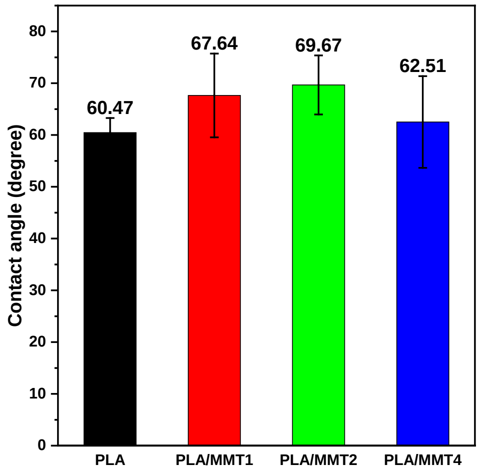

Figure S5. Water contact angle of PLA and PLA/MMT nanocomposites.
